# Enhancing Torque Output for a Magnetic Actuation System for Robotic Spinal Distraction

**DOI:** 10.3390/s25206497

**Published:** 2025-10-21

**Authors:** Yumei Li, Zikang Li, Ding Lu, Tairan Peng, Yunzhi Chen, Gang Fu, Zhenguo Nie, Fangyuan Wei

**Affiliations:** 1 Beijing Key Laboratory of High Dynamic Navigation Technology, Beijing Information Science Technology University, Beijing 100096, China; 20162012@bistu.edu.cn (Y.L.); 2023020436@bistu.edu.cn (Z.L.); ludingcn@163.com (D.L.); 2 Key Laboratory of Modern Measurement Control Technology, Ministry of Education, Beijing Information Science Technology University, Beijing 100096, China; 3 Department of Mechanical Engineering, Tsinghua University, Beijing 100084, China; ptr24@mails.tsinghua.edu.cn; 4 State Key Laboratory of Tribology in Advanced Equipment, Tsinghua University, Beijing 100084, China; 5 Beijing Key Laboratory of Transformative High-End Manufacturing Equipment and Technology, Tsinghua University, Beijing 100084, China; 6 Department of Pediatric Orthopedics, Beijing Jishuitan Hospital, Beijing 100035, China; fugang26@sina.com; 7 Department of Hand and Foot Surgery, Beijing University of Chinese Medicine Third Affiliated Hospital, Beijing 100029, China; footwfy@126.com; 8 Engineering Research Center of Chinese Orthopaedic and Sports Rehabilitation Artificial Intelligent, Ministry of Education, Beijing 100029, China

**Keywords:** early-onset scoliosis, electromagnetic actuator, spinal growing rod, torque optimization

## Abstract

Magnetically controlled spinal growing rods, used for treating early-onset scoliosis (EOS), face a critical clinical limitation: insufficient distraction force. Compounding this issue is the inherent inability to directly monitor the mechanical output of such implants in vivo, which challenges their safety and efficacy. To overcome these limitations, optimizing the rotor’s maximum torque is essential. Furthermore, defining the “continuous rotation domain” establishes a vital safety boundary for stable operation, preventing loss of synchronization and loss of control, thus safeguarding the efficacy of future clinical control strategies. In this study, a transient finite element magnetic field simulation model of a circumferentially distributed permanent magnet–rotor system was established using ANSYS Maxwell (2024). The effects of the clamp angle between the driving magnets and the rotor, the number of pole pairs, the rotor’s outer diameter, and the rotational speed of the driving magnets on the rotor’s maximum torque were systematically analyzed, and the optimized continuous rotation domain of the rotor was determined. The results indicated that when the clamp angle was set at 120°, the number of pole pairs was one, the rotor outer diameter was 8 mm, the rotor achieved its maximum torque and exhibited the largest continuous rotation domain, while the rotational speed of the driving magnets had no effect on maximum torque. Following optimization, the maximum torque of the simulation increased by 201% compared with the pre-optimization condition, and the continuous rotation domain was significantly enlarged. To validate the simulation, a rotor torque measurement setup incorporating a torque sensor was constructed. Experimental results showed that the maximum torque improved from 30 N·mm before optimization to 90 N·mm after optimization, while the driving magnets maintained stable rotation throughout the process. Furthermore, a spinal growing rod test platform equipped with a pressure sensor was developed to evaluate actuator performance and measure the maximum distraction force. The optimized growing rod achieved a peak distraction force of 413 N, nearly double that of the commercial MAGEC system, which reached only 208 N. The simulation and experimental methodologies established in this study not only optimizes the device’s performance but also provides a viable pathway for in vivo performance prediction and monitoring, addressing a critical need in smart implantable technology.

## 1. Introduction

Early-onset scoliosis (EOS) is a severe spinal deformity that significantly impairs cardiopulmonary development, musculoskeletal balance, and psychosocial health in pediatric patients. As the condition progresses, it disrupts normal pelvic kinematics and spinal alignment, often resulting in progressive deformities. In untreated or severe cases, EOS has been reported to cause cardiopulmonary insufficiency and, in extreme scenarios, cardiopulmonary failure [1,2].

When curvature progression exceeds the limits of conservative management, surgical intervention with expandable growth rods is required to control deformity progression and preserve thoracic development. Among the surgical strategies, the Magnetically Controlled Growth Rod (MAGEC) system, developed by NuVasive, has demonstrated favorable outcomes in the management of EOS. This medical robotic system comprises an external magnetic actuator and an implantable rod. The actuator employs a pair of permanent drive magnets to produce a rotating magnetic field, which induces rotation of an internal rotor within the rod, generating torque. This torque is subsequently transmitted through a gearing system and converted into axial displacement, enabling the rod to lengthen or retract. The MAGEC rod is capable of achieving a maximum traction force of approximately 208 N [3,4,5,6,7]. Despite these promising results, clinical studies have consistently reported a recurring limitation: the traction force generated by the device is often insufficient [7]. This limitation can result in unsuccessful lengthening procedures, compromised spinal correction, and ultimately suboptimal therapeutic outcomes. Overcoming this limitation requires enhancing the maximum torque output of the rotor and expanding the continuous rotation domain—the spatial region within which the rotor maintains synchronous rotation with the actuator. The maximum rotor torque directly determines the traction force of the growth rod; higher torque output enables greater traction force. Moreover, variations in patient anthropometrics and actuator positioning across clinical cases make an expanded continuous rotation domain essential for ensuring stable and reliable device performance in diverse populations.

The commercial MAGEC system—an archetypal magnetically controlled growth rod (MCGR) for EOS treatment—has robust systematic evidence supporting its efficacy, complication profile, and magnetic control adaptability, with well-documented dimensional specifications including 4.5 mm/5 mm rod diameters, 70 mm/90 mm rod lengths, and standard/offset configurations for dual-rod independent distraction, plus a non-contourable actuator clinically required to be placed over the straight thoracolumbar spine and secured via pedicle screws, hooks, or rib fixation [8,9,10,11,12]. Clinically, it delivers therapeutic benefits through non-invasive serial lengthening and reduces planned reoperations versus traditional growth rods (TGRs), yet it fails to eliminate the “law of diminishing returns” and is associated with dual complications, including anchor failures, mechanical malfunctions, postoperative infections, and rare long-term spontaneous fusion. Collectively, these data confirm MAGEC’s benchmark role in EOS management but highlight critical limitations—inadequate torque transmission and structural safety concerns—which directly align with the earlier clinical challenges of insufficient traction force.

Parallel to research on spinal instrumentation, significant research on torque optimization in permanent magnet (PM) motors offers valuable insights for magnetic actuator design. Studies can be broadly categorized into control strategy improvements and motor structure innovations. For instance, Sun et al. [13] optimized the stator core geometry and air-gap length in permanent magnet synchronous motors (PMSMs), achieving a 24% reduction in torque ripple. Zhou et al. [14] developed a multi-objective optimization framework incorporating genetic algorithms to minimize torque ripple and enhance torque output under representative electric vehicle operating conditions. Their results showed a 33.5% reduction in torque ripple and a 7.84% increase in torque under heavy-load scenarios, along with a 3.6% gain during high-speed cruising. Shi et al. [15] applied finite element analysis (FEA) to examine cogging torque and dynamic performance under different skew angles, identifying an optimal configuration through mathematical optimization. Similarly, Bingi et al. [16] introduced a neural network–based torque prediction model capable of accurately predicting torque without the need for additional sensors.

Other researchers have focused on structural innovations to enhance PM motor performance. Güemes et al. [17] evaluated the influence of slot number in 20-pole PMSMs and demonstrated that fractional-slot windings reduced cogging torque and torque ripple, although at the expense of average torque. Usman et al. [18] proposed hexagonal arc-shaped permanent magnets combined with asymmetric overhang optimization, employing three-dimensional FEA, Latin hypercube sampling, Kriging models, and genetic algorithms. Their approach achieved notable reductions in cogging torque and improvements in axial flux PM motor performance. Ebrahimi et al. [19] introduced a novel PM motor topology, derived an analytical model of cogging torque, and validated it with FEA, achieving significant improvements in cogging torque suppression, average electromagnetic torque, torque density, and peak torque. Yan et al. [20] investigated magnetic pole-edge effects by decomposing cogging torque into amplitude and phase offsets and optimized segmented pole lengths to minimize torque ripple. Liu et al. [21] systematically compared four pole-slot ratio schemes for a 37 kW, 160 rpm low-speed, high-torque PM motor. By combining two sampling methods with three surrogate modeling approaches, they constructed six models and validated the optimized design experimentally, providing reliable reference data for both initial and optimized motor designs. Nobahari et al. [22] designed an axially segmented Interior Permanent Magnet–Synchronous Reluctance Motor (IPM-SyRM) optimized using a computationally efficient algorithm for lightweight electric vehicle applications, demonstrating superior performance over conventional designs through prototyping and testing.

Collectively, these studies have enriched the theoretical and practical understanding of torque optimization in PM motors. They consistently show that structural refinements, numerical modeling, and surrogate-based optimization methods can effectively improve performance by reducing torque ripple and increasing torque output. However, within the context of implantable medical robotic systems such as magnetically controlled growth rods, few efforts have directly addressed actuator torque optimization—a factor crucial to achieving reliable mechanical elongation and improved clinical utility in the treatment of early-onset scoliosis. The primary aim of this study is to optimize the torque output of the magnetic actuator and define the continuous rotation domain of the rotor for magnetically controlled spinal growing rods. Critically, the models and performance data generated in this work serve as essential prerequisites for integrating sensor-based, real-time force monitoring and closed-loop control strategies in next-generation smart implantable devices. To this end, a finite-element-based dynamic magnetic field simulation model for a circumferentially distributed permanent magnet-rotor drive is established using ANSYS-Maxwell. This model systematically investigates the influences of four key parameters—the clamp angle between the driving magnet and the rotor, the number of pole pairs, the outer diameter of the rotor, and the rotational speed of the driving magnet—on the maximum torque and the continuous rotation range of the rotor. To rigorously validate the simulation results and provide reliable experimental data, a dedicated rotor torque measurement device, incorporating a high-precision torque sensor, and a spinal growing rod test platform, equipped with a force sensor, were designed and fabricated. These sensor-instrumented setups were essential for direct performance quantification, bridging high-fidelity simulation with physical validation and laying the groundwork for future intelligent implant systems.

## 2. Theoretical Model

### 2.1. Overview of the MAGEC System for Spinal Growing Rod

**Figure 1:**
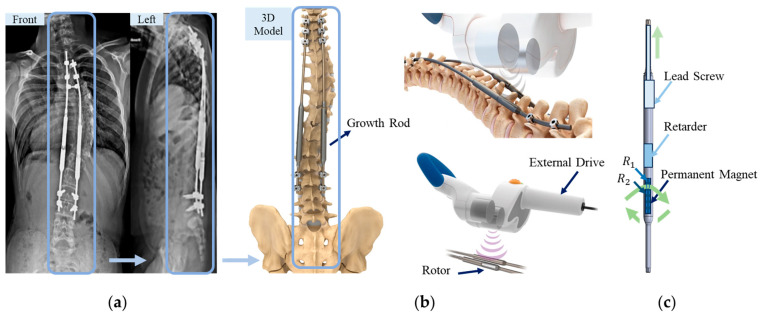
Schematic of the magnetically controlled growth rod system: (**a**) X-ray image of a spine with dual implanted rods [3]; (**b**) External magnetic actuator with dual rotating permanent magnets; (**c**) Schematic of the internal rod structure and actuation mechanism. Here, $R_{1}$ and $R_{2}$ represent the inner diameter and outer diameter of the rotor respectively.

When EOS becomes severe and continues to progress, surgical intervention with single or dual expandable growth rods is typically required. These rods are anchored to the upper thoracic and lower lumbar regions of the spine to provide corrective traction along the spinal axis. Among various configurations, dual-rod constructs offer superior biomechanical stability and greater deformity correction, thereby reducing the likelihood of rod fracture or implant failure. The MAGEC system allows non-invasive adjustment of spinal rods through an external magnetic actuator. Figure 1a shows an X-ray of a patient with dual magnetically controlled growing rods. Figure 1b depicts the external magnetic drive system, which incorporates two identical, axially rotating permanent magnets that generate a rotating magnetic field. This field induces torque in a rotor located within the implanted rod. The torque is amplified through a mechanical conversion mechanism and translated into axial displacement, enabling the rod to either elongate or retract, as schematically illustrated in Figure 1c.

In clinical practice, the MAGEC system reduces the frequency of repeated surgeries and related complications by facilitating regular non-invasive lengthening procedures. However, anatomical variability among patients, such as differences in soft tissue thickness and spinal curvature, can lead to suboptimal magnetic coupling between the external actuator and the internal rotor. This misalignment may result in inconsistent lengthening outcomes, insufficient traction force, and unreliable actuation. Therefore, a thorough understanding of the physical principles and governing equations of magnetic torque generation is essential to overcome these limitations and guide the design optimization of the system.

### 2.2. Mathematical Formulation of Magnetic Components

The driving permanent magnets and the rotor in the MAGEC system are cylindrical in shape. The magnetic force generated by a cylindrical permanent magnet can be expressed based on its magnetic field intensity and geometric parameters. The magnetization intensity *M* relates to the magnetic field intensity *H* and the magnetic flux density *B*:

(1) $$B=\mu_{0}\left(H+M\right)$$

where $\mu_{0}$ is the permeability of free space.

The magnetic moment μ of the magnet depends on its magnetization intensity and geometry:

(2) $$\mu =M\cdot \pi R^{2}L$$

where R is the radius and L is the length of the cylindrical magnet.

The magnetic force F acting on the rotor is related to the field intensity and magnetic moment:

(3) $$F=\frac{3\mu_{0}\mu^{2}}{2\pi r^{4}}$$

where r is the center-to-center distance between the rotor and the external magnet.

The basic equation for calculating the torque T on a cylindrical rotor is as follows:

(4) $$T=k_{T}\cdot \mu \cdot B$$

where $k_{T}$ is a torque coefficient. This shows that rotor torque depends on the magnetization intensities, dimensions of the rotor and external magnets, and their relative distance.

### 2.3. Governing Equation

Because no current flows in the magnetic field generated by permanent magnets, the “Magnetostatic, No Current” module in ANSYS Maxwell was employed for simulation. The governing equation is as follows:

(5) $$\nabla \cdot \left(\mu_{r}\nabla U\right)=0$$

where U is the scalar magnetic potential and $\mu_{r}$ is the material permeability.

### 2.4. Rotor Moment of Inertia

The moment of inertia defines an object’s resistance to angular acceleration. To simulate rotor dynamics under magnetic actuation, it is necessary to calculate the rotor’s moment of inertia. For a cylindrical rotor with inner radius $R_{1}$, outer radius $R_{2}$, height h, and material density ρ, the mass m is as follows:

(6) $$m=\rho \pi \left({R_{2}}^{2}-{R_{1}}^{2}\right)h$$

The moment of inertia about its symmetry axis is as follows:

(7) $$I=\frac{1}{2}m\left({R_{1}}^{2}+{R_{2}}^{2}\right)$$

## 3. Magnetically Controlled Growth Bar System and Driven Permanent Magnet-Rotor Modeling

### 3.1. 3D Model Description

**Figure 2:**
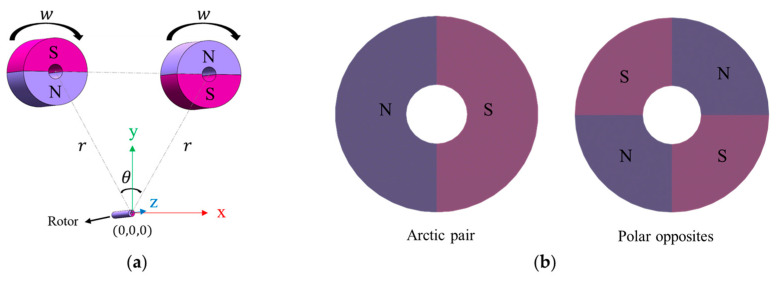
Models of the permanent magnet–rotor system: (**a**) Finite element model constructed in ANSYS Maxwell; (**b**) Schematic diagram illustrating pole pairs in a permanent magnet.

A finite element model (FEM) of this system was constructed using ANSYS Maxwell, as shown in Figure 2a. Both the rotor and the driving magnets were set to a length of 40 mm and placed in the same horizontal plane. The rotor axis was aligned with the *z*-axis, and the x–y plane was defined at one end of the rotor, with the coordinate origin located at its center.

The angle θ in the x–y plane was defined as the angle between the rotor center and the centers of the two driving magnets. This angle plays a critical role in determining the effectiveness of magnetic coupling and the resulting torque output. A parameterized geometric model was adopted to allow systematic variation of key dimensions and configurations during simulation.

### 3.2. Magnetic Configuration and Simulation Domain

Both the rotor and the driving magnets were radially magnetized. The number of poles was set to twice the number of pole pairs, ensuring symmetrical pole distribution. Radial magnetization directs the magnetic field lines outward from the magnet center, promoting efficient interaction with the rotor.

Since the implanted growth rod is surrounded by biological soft tissue that exhibits magnetic permeability close to that of air or vacuum, the external simulation domain was modeled as a vacuum. This assumption simplifies the analysis without compromising accuracy.

### 3.3. Model Parameters and Material Properties

Table 1 summarizes the geometric parameters of the rotor and the driving magnets, which were used to calculate the rotor’s moment of inertia. Based on these values, the rotor moment of inertia was calculated as 1.769 × 10^−8^ kg·m^2^.

The driving magnets and rotor were composed of neodymium–iron–boron (NdFeB), a clinically applicable magnetic material for implantable spinal devices. It is characterized by a magnetic energy product that balances magnetic performance and long-term stability, and its core magnetic properties—including remanent flux density, coercivity, and Curie temperature—meet the requirements of spinal growing rod implantation. Specifically, within the human body temperature range, the remanence attenuation rate of NdFeB grade 35 is less than 1.2% per year, and it maintains stable magnetic behavior within the operating temperature range of −40~80 °C. The relevant material properties are listed in Table 2; these parameters were fully input into the finite element model (FEM) of ANSYS Maxwell to ensure accurate simulation of the magnets’ magnetic field distribution and torque generation characteristics. NdFeB grade 35 is selected for spinal growing rods due to its balanced magnetic performance ((BH) max = 35 MGOe, meeting the ≥90 N·mm post-optimization torque requirement) and clinical safety: it complies with ISO 10993-1 [23] biocompatibility standards, shows no tissue injury in cadaveric studies, and maintains stability under 1.5T MRI (max tissue temperature rise 3.6 °C, force output within 10% of baseline)—critical for long-term (≥5 years) pediatric implantation and adapting to EOS patients’ need for recurrent MRI [24,25]. The symbol and definition of the simulation parameters for the finite element model are presented in Appendix A Table A1.

## 4. Simulation Results and Parameter Optimization Analysis

Using the parameterized model in ANSYS Maxwell, three-dimensional simulations were conducted with both the rotor and driving magnets set to a length of 40 mm. Parameter sweeps were performed for rotational speed w, rotor outer diameter q, angular position θ, number of pole pairs s, and rotor position $\left(x,y\right)$. The objective was to evaluate the maximum torque of the rotor under different conditions and identify the optimal configuration for continuous rotation.

### 4.1. Maximum Rotor Torque

**Figure 3:**
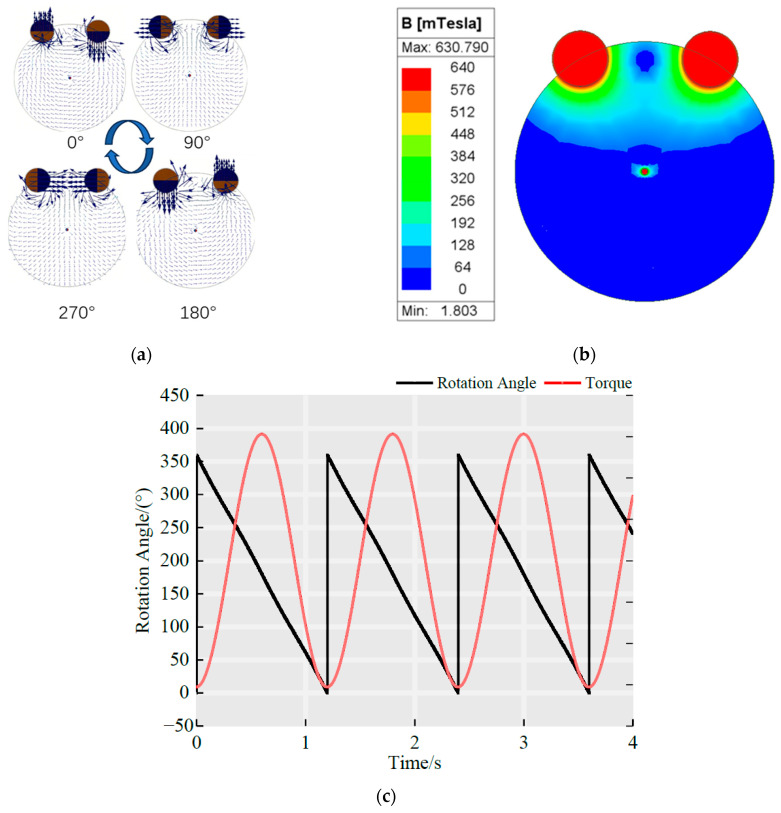
Simulation results of the driving permanent magnet-rotor model: (**a**) Distribution of magnetic field lines; (**b**) Distribution of magnetic field intensity; (**c**) Time-dependent variation of rotor torque.

Under the conditions q =5 mm, s=1, and θ=60°, the rotor was fixed while the driving magnets rotated at a speed of 50 r/min. The corresponding magnetic field lines and intensity distributions are shown in Figure 3a,b. The time-dependent rotor torque exhibited a sinusoidal pattern, with the peak value representing the maximum torque, as illustrated in Figure 3c. Under these conditions, the maximum torque reached 32.847 N·mm, and the rotor rotated synchronously with the magnets.

**Figure 4:**
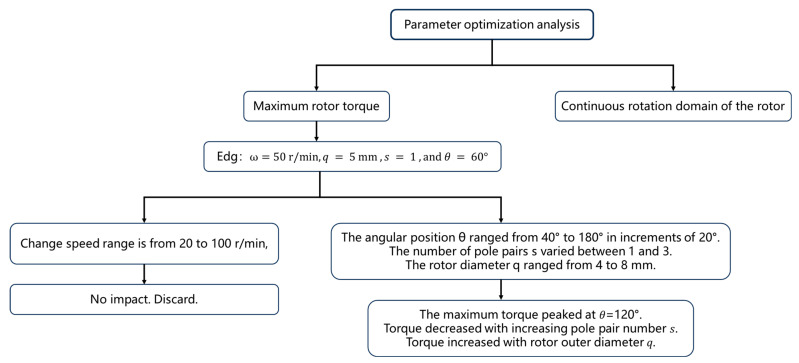
Optimization flowchart of the optimization method.

**Figure 5:**
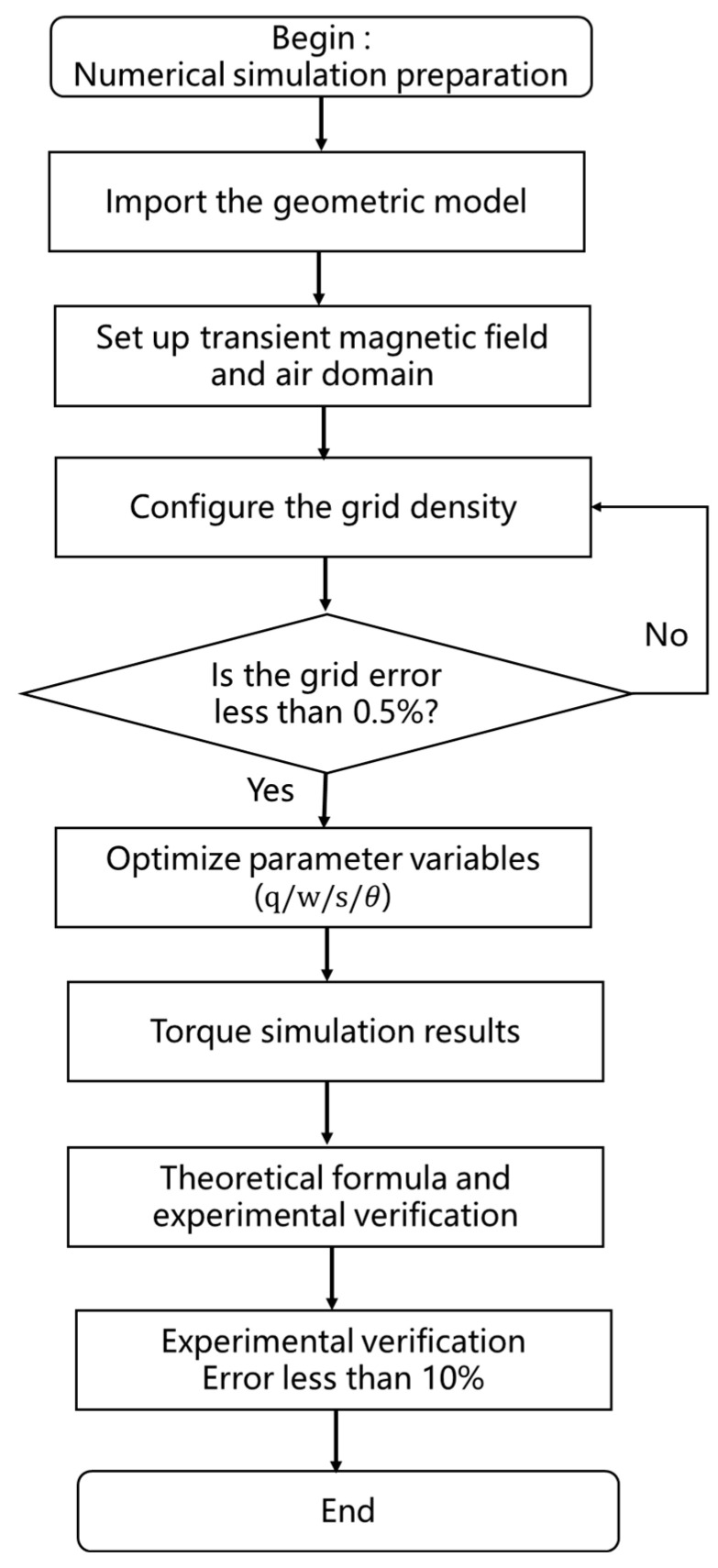
Flowchart of the numerical simulation process.

**Figure 6:**
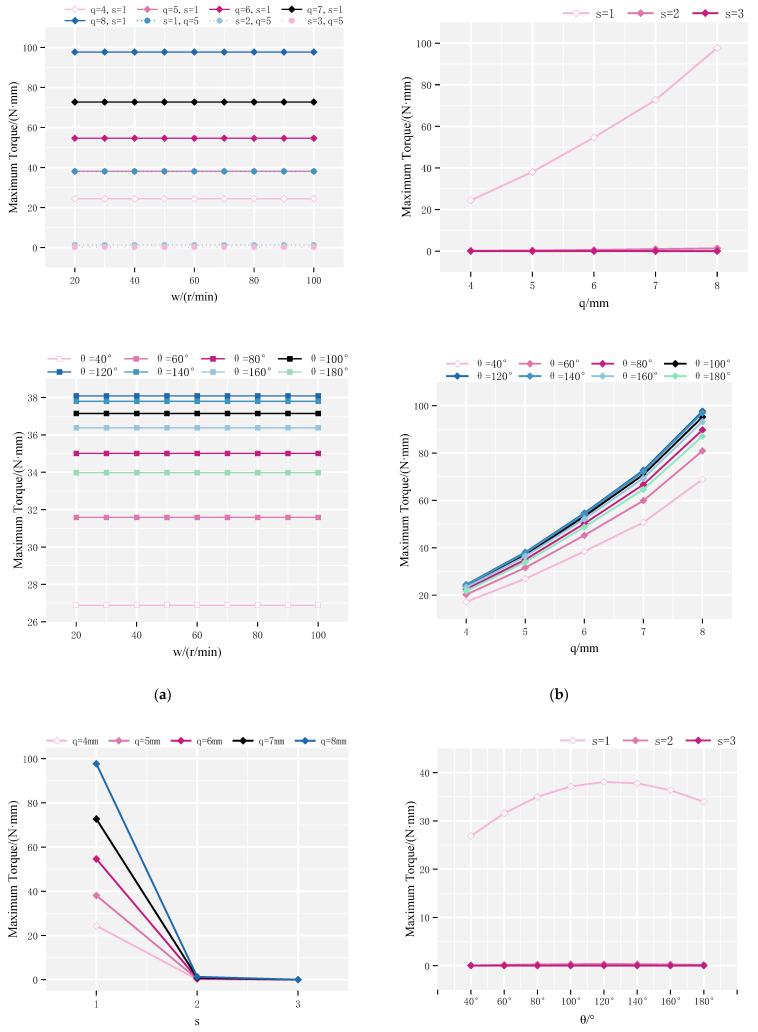

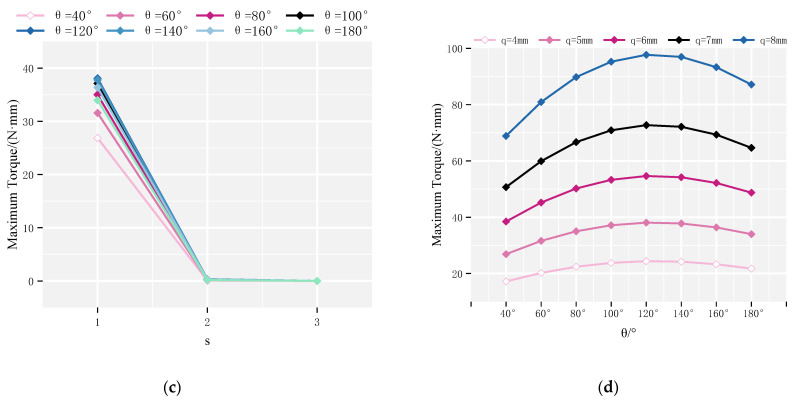
Effects of design parameters on maximum rotor torque: (**a**) Torque variation with w under different q, s and θ; (**b**) Torque variation with s under different q and θ; (**c**) Torque variation with q under different s and θ; (**d**) Torque variation with θ under different q and s.

Further simulations were performed to analyze the influence of different design parameters. Among them, the optimization process of the optimization method is shown in Figure 4, and the process of the numerical simulation is shown in Figure 5. The angular position θ ranged from 40° to 180° in increments of 20°. The number of pole pairs s varied between 1 and 3, the rotor diameter q ranged from 4 to 8 mm, and the rotation speed ω was varied from 20 to 100 r/min. Results, shown in Figure 6, revealed that: The maximum torque peaked at θ=120°. Torque decreased with increasing pole pair number s. Torque increased with rotor outer diameter q. Torque was largely unaffected by changes in rotation speed s.

### 4.2. Continuous Rotation Domain of the Rotor

The spatial domain in which the rotor can achieve continuous, synchronous rotation with the external magnetic field is clinically significant, particularly given variability in patient anatomy and implant positioning. In typical MAGEC system implantation, one end of the rod is anchored at the T6–T8 vertebrae and the other at the L4–L5 vertebrae, which may lead to variations in the relative alignment of the internal rotor and external actuator.

To evaluate the effective rotation domain, simulations were performed under the following conditions: angular position θ=120°, rotor outer diameter q=8 mm, rotor length = 60 mm, rotation speed w=50 r/min, and pole pair number s=1. The rotor’s x–y coordinate boundaries were set to [−10,10] mm and [−10,10] mm, respectively, while the z-coordinate remained fixed. These limits represent clinically plausible positions for magnetic interaction.

**Figure 7:**
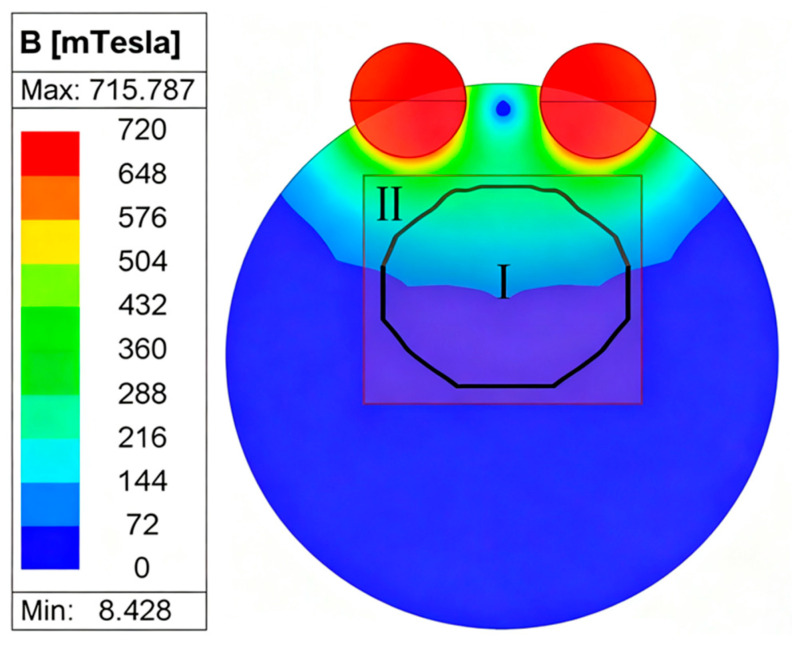
Continuous rotation domain of the optimized rotor configuration.

Simulation results divided the domain into two distinct regions: Zone I (Synchronous Region): The rotor maintains continuous synchronous rotation with the driving magnets. Zone II (Non-synchronous Region): The magnetic torque is insufficient to sustain synchronous rotation. As illustrated in Figure 7, the optimized configuration significantly expanded Zone I, thereby enhancing the adaptability and clinical efficacy of the MAGEC system. Expansion of the synchronous domain ensures stable magnetic coupling even under suboptimal alignment during implantation or patient movement.

### 4.3. Optimization Analysis

**Figure 8:**
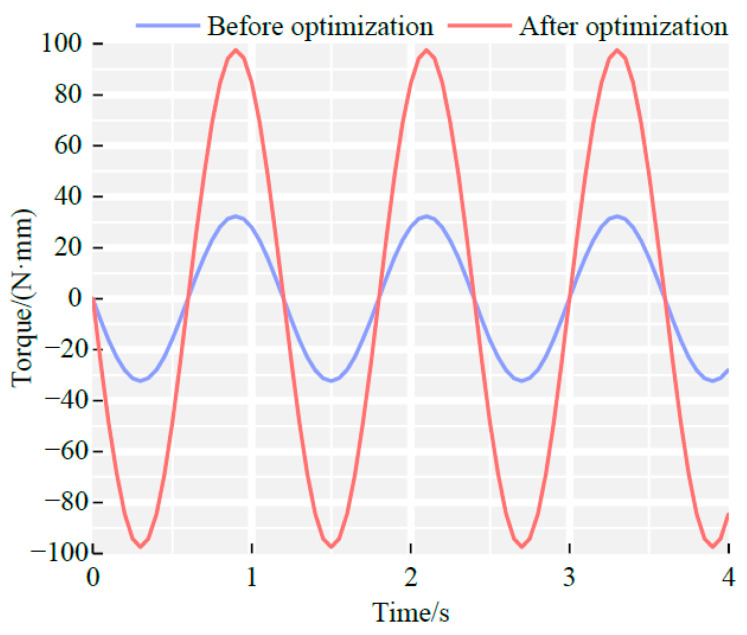
Comparison of rotor torque variation before and after optimization.

To quantitatively assess the improvement achieved by parameter optimization, a comparative torque analysis was performed before and after optimization, as shown in Figure 8. The optimized configuration was defined as: θ = 120°, s = 1, q = 8 mm, and w = 50 r/min. All other simulations were held constant. As shown in Figure 6, the maximum torque of the pre-optimization rotor was 32.847 N·mm, while the optimized rotor achieved a maximum torque of 98.970 N·mm, representing a 201% increase.

## 5. Experimental Verification of Magnetron Drives

**Figure 9:**
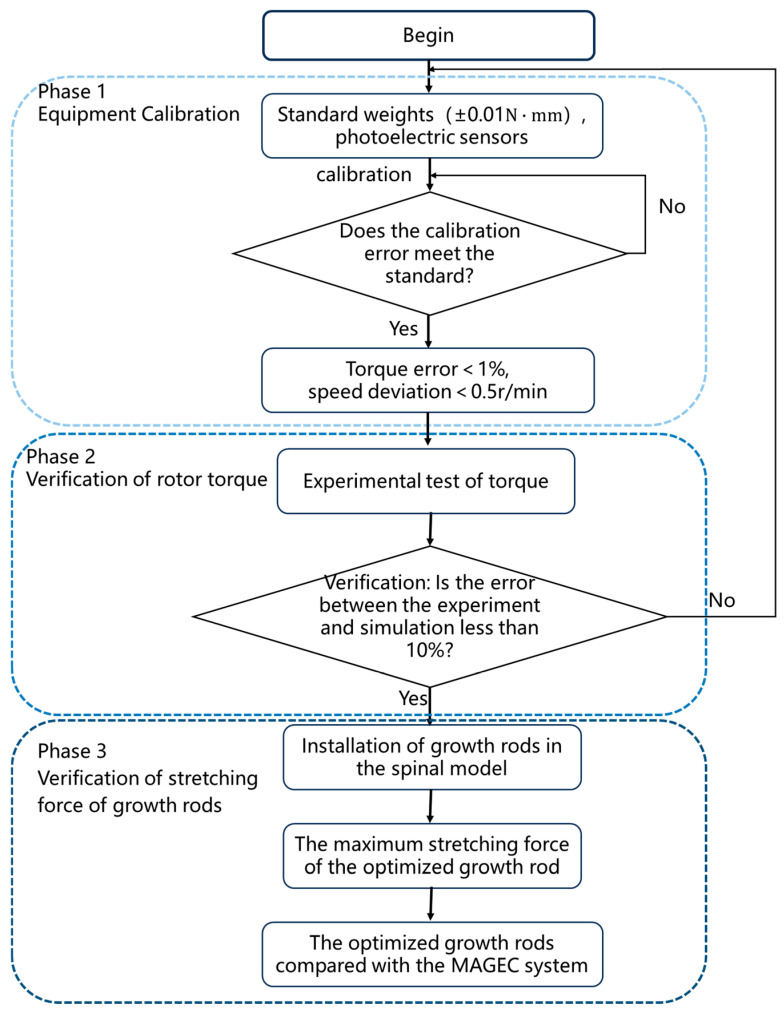
Experimental design logic flowchart.

**Figure 10:**
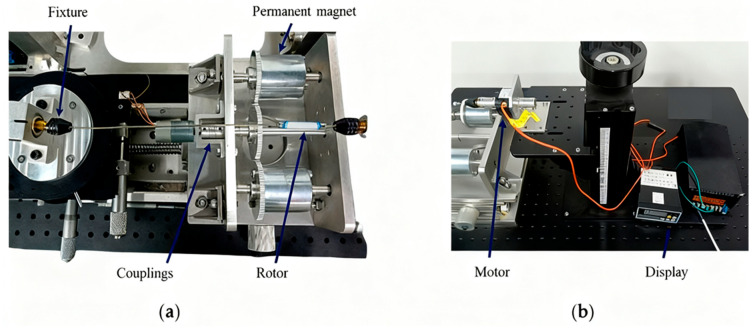
Rotor torque measuring device: (**a**) Drive device; (**b**) Measuring device.

The flowchart of the experimental design is shown in Figure 9.To validate the simulation results, a rotor torque testing system was developed, as shown in Figure 10. In this setup, a motor was connected to a transmission shaft through a flexible coupling. The opposite end of the shaft was bolted to a gear assembly, which synchronously drove two permanent magnets via meshed gears—this configuration ensured that the magnets rotated in unison, thereby replicating the actuation conditions of magnetic control systems. A torque sensor was used to directly measure the output torque of the rotor; the sensor was mounted securely with a combination of set screws, locking blocks, and high-rigidity couplings to minimize mechanical interference. The rotor, screw rod, and collet were all fixed to the torque sensor through coaxial couplings, ensuring concentricity between the rotor and the sensor to guarantee stable and high-precision data acquisition. This system allowed direct, real-time measurement of rotor torque under both pre- and post-optimization configurations. Notably, the torque data obtained from this high-accuracy torque sensor served as the gold standard for validating the reliability of the ANSYS Maxwell simulation model, as it provided objective, in vitro measurement results that directly reflected the actual torque output of the magnetic actuation system.

Experimental results confirmed the effectiveness of the optimized design. Prior to optimization, the maximum measured torque was 30 N·mm; after optimization, the maximum torque increased to 90 N·mm. The corresponding errors between experimental and simulated results were 8.7% and 9.06%, respectively, with both systems maintaining stable magnet rotation.

**Figure 11:**
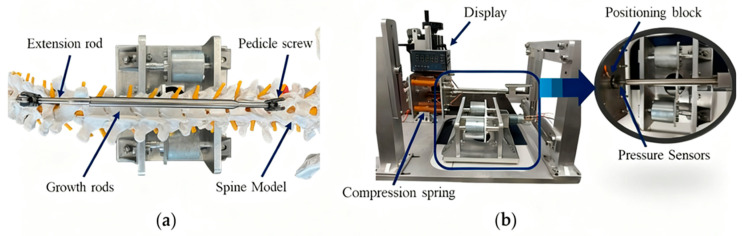
Growing rod experimental apparatus: (**a**) Growing rod extension setup; (**b**) Distraction force measurement test rig and pressure sensor module for distraction experiments.

To further evaluate actuator performance under load, a growth rod experimental apparatus was assembled (Figure 11a). Pedicle screws were used to anchor both ends of the magnetic growth rod onto a spinal model; the rod consisted of several interconnected components: a permanent magnet rotor, a reducer, a lead screw, and an extension rod. When the external actuator generated a rotating magnetic field, it induced rotor rotation, which was amplified by the reducer and transmitted to the lead screw—this torque was then converted into linear axial elongation. A separate test rig (Figure 11b) was constructed to measure the maximum distraction force of the system: in this setup, one end of the growth rod was fixed, while the other end pressed against a sensor positioning block integrated with a load cell. The compression of the spring system was translated into an elongation force, which was directly and precisely measured by the load cell and recorded in real time via a data acquisition module. The distraction force data obtained from this load cell served as the gold standard for validating the simulation model’s prediction of the actuation system’s clinical performance, as it accurately replicated the in vivo force transmission scenario of spinal distraction and provided quantitative evidence for the optimized system’s efficacy.

Results showed that the optimized growth rod achieved a peak distraction force of 413 ± 18 N (mean ± standard deviation) over five independent tests—nearly double that of the commercial MAGEC system (208 N) [4].

## 6. Conclusions

This study focuses on optimizing the permanent magnet drive system of magnetically controlled spinal growing rods to address the clinical limitation of insufficient distraction force in EOS treatment. A transient finite element magnetic field simulation model of a circumferentially distributed permanent magnet–rotor system was established using ANSYS Maxwell to systematically investigate the effects of four key parameters—clamp angle between driving magnets and the rotor (θ), number of magnetic pole pairs (s), rotor outer diameter (q), and driving magnet rotational speed (w)—on rotor maximum torque, while defining the parameter space for the optimized rotor’s stable continuous rotation. Crucially, experimental validation was performed using custom-built platforms instrumented with precise sensors: a rotor torque measurement apparatus and a spinal growing rod testing system, which directly quantified performance metrics. The results confirmed an optimal configuration (clamp angle θ = 120°, one pole pair, rotor diameter q = 8 mm), leading to a 201% improvement in torque and a peak distraction force of 413 N—nearly double that of the commercial MAGEC system.

Limitations of the study include the vacuum assumption in magnetic field simulations (neglecting dynamic soft tissue magnetic permeability changes), restricted speed analysis to 20–100 r/min (excluding high-speed eddy current effects), simplified soft tissue resistance simulation via a spring (failing to replicate viscoelasticity), and unevaluated interference from additional metal implants. Clinically, the optimized system is suitable for 2–15-year-old EOS patients with progressive curvature (Cobb angle > 20°, annual progression > 5°) unresponsive to conservative treatment, no ferromagnetic implants (e.g., pacemakers), and normal bone density (T-score > −1.0), designed for dual-rod anchorage at T6–T8 and L4–L5, while being inapplicable to severe deformities (Cobb angle > 45°), implantation exceeding 8 years, or magnetic resonance imaging (MRI) with field strengths > 3.0T. By integrating sensor-based experimental validation with numerical optimization, this work not only provides a reliable strategy for enhancing actuator performance but also establishes a measurable foundation for future developments in smart implants capable of real-time force monitoring. Overall, the findings demonstrate significant potential to overcome the insufficient distraction force observed in current clinical devices.

## Figures and Tables

**Table 1:** Geometric specifications of the driving permanent magnets and rotors.

Parameters	Drive Permanent Magnets	Rotors
Inside diameter/$d\;\left(mm\right)$	11	2
External diameter/D (mm)	50	5
Thicknesses/t (mm)	40	40

**Table 2:** Material properties of NdFeB permanent magnets.

Parameters	Numerical Value
Magnetic field strength/$H_{c}$ $(A\cdot m^{-1}$)	$8.90\times 10^{5}$
Relative permeability/$\mu_{r}$	1.100
Electrical conductivity/$\sigma \;\left(s\cdot m^{-1}\right)$	$6.250\times 10^{5}$
Density/$\rho \left(kg\cdot m^{-3}\right)$	$7.40\times 10^{3}$
Remanent Flux Density/$B_{r\;}$(T)	1.30
Operating Temperature Range	−40~80 °C
Curie Temperature/$T_{c}$ (°C)	310 °C
Magnet Grade	NdFeB 35
Maximum Energy Product/(BH)max	35 MGOe

## Data Availability

The data that support the findings of this study are available from the corresponding author upon reasonable request.

## Abbreviations

The following abbreviations are used in this manuscript:

MAGEC	MAGnetic Expansion Control
EOS	Early-onset scoliosis
PM	permanent magnet
PMSMs	permanent magnet synchronous motors
FEA	finite element analysis
FEM	finite element model

## Appendix A

Table A1 provides the simulation parameters and their symbols used throughout the analysis. Parameters include magnetic field strength, angular velocity, and rotor–magnet spacing. Each variable was systematically varied to evaluate its influence on torque generation and dynamic performance.

**Table A1:** Finite element model simulation parameters and definitions.

Parameter	Definition
$\omega (r\cdot {min}^{-1})$	Angular velocity of the driving magnet
$q\left(mm\right)$	Rotor outer diameter
$a\left(mm\right)$	Outer diameter of driving magnet
$\theta \left({}^{\circ}\right)$	Angle between driving magnet and rotor
s	Number of pole pairs
$x,y\left(mm\right)$	Coordinates on rotor end face
$r\left(mm\right)$	Center-to-center distance (x–y plane)
